# Pharmaceutical applications of lignin-derived chemicals and lignin-based materials: linking lignin source and processing with clinical indication

**DOI:** 10.1007/s13399-023-03745-5

**Published:** 2023-01-21

**Authors:** Pinar Karagoz, Sansanee Khiawjan, Marco P. C. Marques, Samir Santzouk, Timothy D. H. Bugg, Gary J. Lye

**Affiliations:** 1 Department of Biochemical Engineering, The Advanced Centre for Biochemical Engineering, University College London, Gower Street, London, WC1E 6BT UK; 2 School of Engineering and Innovation, The Open University, Milton Keynes, MK7 6AA UK; 3 Panax-Homeopathy and Phytotherapy Laboratory, Agrinio, Greece; 4 Department of Chemistry, University of Warwick, Coventry, CV4 7AL UK

**Keywords:** Lignocellulose biomass, Lignin, Pharmaceuticals, Drug delivery

## Abstract

Lignocellulosic biomass is one of the most abundant bioresources on Earth. Over recent decades, various valorisation techniques have been developed to produce value-added products from the cellulosic and hemicellulosic fractions of this biomass. Lignin is the third major component accounting for 10–30% (w/w). However, it currently remains a largely unused fraction due to its recalcitrance and complex structure. The increase in the global demand for lignocellulosic biomass, for energy and chemical production, is increasing the amount of waste lignin available. Approaches to date for valorizing this renewable but heterogeneous chemical resource have mainly focused on production of materials and fine chemicals. Greater value could be gained by developing higher value pharmaceutical applications which would help to improve integrated biorefinery economics. In this review, different lignin extraction methods, such as organosolv and ionic liquid, and the properties and potential of the extracted chemical building blocks are first summarized with respect to pharmaceutical use. The review then discusses the many recent advances made regarding the medical or therapeutic potential of lignin-derived materials such as antimicrobial, antiviral, and antitumor compounds and in controlled drug delivery. The aim is to draw out the link between the source and the processing of the biomass and potential clinical applications. We then highlight four key areas for future research if therapeutic applications of lignin-derived products are to become commercially viable. These relate to the availability and processing of lignocellulosic biomass, technologies for the purification of specific compounds, enhancements in process yield, and progression to human clinical trials.

## Introduction

Lignin is the second most abundant biopolymer after cellulose, accounting for approximately 10–30% (w/w) of the organic carbon in the biosphere [1]. Lignocellulosic biomass has attracted widespread attention for energy production, in particular bioethanol generation, due to its high cellulose content. Lignin, a major component of lignocellulosic biomass, does not contain carbohydrates and is not usable for bioethanol production [2]. It has thus been viewed as an unwanted waste material or low value by-product that is best burnt to generate electricity or heat [3].

In recent years, global bioethanol production has increased to meet the world’s energy demand and reduce greenhouse gas emissions caused by fossil fuel use [4]. It is estimated by 2030, as annual biofuel production in the United States (US) grows to 60 billion gallons, that production of lignin in US refineries alone will reach 0.225 billion tonnes [5, 6]. Valorisation of lignin post bioethanol synthesis has been a focus of research for cost-effective biofuel production [7]. The chemical heterogeneity of lignin has been a particular challenge limiting its use. However, there are now multiple studies showing the feasibility of extracting and synthesizing high-value products from lignin, and these are reviewed in this article.

Widespread advances in science and technology have underpinned the growth of the pharmaceutical industry. Today people can expect to live longer and better than they did a century ago. Furthermore, the pharmaceutical industry plays a critical role in the global economy. In 2017 the industry invested nearly € 35,200 on R&D in Europe alone [8]. However, it is estimated that around one-third of the world’s population still has no access to essential medicines [9]. Lowering the raw material and production costs of new medicines can increase the affordability and availability of new drugs. Lignocellulosic materials thus have significant potential to enable sustainable economic growth of the pharmaceutical sector.

Cellulose and its derivatives, including cellulose esters, already find widespread use in drug delivery systems [10]. Use of innovative cellulose-based materials, including micro-cellulose (MC) and nano-cellulose (NC) for sustained drug delivery, has attracted considerable interest [11]. Oxycellulose (OC or oxidized cellulose) is another cellulose derivative widely used as a tablet excipient [12]. Similarly, hemicellulose-based materials have been used to develop new drug delivery systems [13], antithrombotic agents [14], and cancer therapeutics [15]. For example, hemicellulose extracted from spent liquor was utilized to develop hydrogels that can rapidly respond to pH and salt concentration and be applied for controlled drug release [16]. Furthermore, xylose (the main sugar present in hemicellulose) is widely used as a low calorie sweetener for diabetes and can also promote proliferation of human intestinal *Bifidobacteria* to help enhance immunity [17].

Alongside these other lignocellulosic biopolymers, lignin has the potential to replace diminishing fossil-based resources for the sustainable production of various chemicals [18]. Its derivatives are already understood to be essential components present in traditional medicine; hence, it could play an increasingly important role in development of value-added pharmaceuticals [19]. Valorisation of lignin in this way could also have a positive impact on the future development of biorefineries and the bio-based economy. The main pharmacological activities of lignin can be categorized as (i) antioxidant activity and protection against oxidative stress, (ii) antimutagenic and antitumor activity, (iii) antiviral and antimicrobial activity, (iv) immunomodulatory effects, and (v) intestinal activities (such as anti-diarrheal effects) [20]. One of the most promising uses of lignin compounds stems from their antioxidant capacity [21]. Antioxidants are compounds that delay autoxidation by inhibiting formation of free radicals or by interrupting propagation of the free radicals [22]. Furthermore, recent studies showed that lignin-based hydrogels have great potential for developing new drug delivery systems [23]. At present, however, the complex and variable structure of lignin according to its origin, separation, and purification still limits commercial applications [24].

This work provides a comprehensive review of the pharmaceutical and healthcare potential of lignin-based chemicals and materials. Previously, the pharmaceutical benefits of lignin and its derivatives were described in [25]. That study provided information from articles published between 2010 and 2016. Here, we present recent findings including on new and greener lignin extraction methods, and we systematically categorize lignin types and their pharmaceutical potential according to their chemical structures. We discuss current strategies for developing novel lignin-based hydrogels and studies using lignin-carbohydrate complexes for different pharmaceutical purposes, and we highlighted the potential of lignophenols. We believe that this review will provide a valuable perspective that helps endorse further research on lignin-based pharmaceutical production.

## Source and structure of lignin

Lignin, a three-dimensional amorphous polymer, is one of the three major components of lignocellulosic biomass [26], and the amount of lignin varies depending on the source (Table 1). Agro‐industrial activities generate large amounts of lignocellulosic wastes (non-edible parts of edible plants and/or non-edible plants) such as straw, bagasse, foliage, bunches, and shells. A proportion of these discarded materials are removed during the first stage of the harvesting process and are either left in the field, used as animal feed, or incinerated despite their rich carbohydrate content [27].

**Table 1:** Renewable forestry and agricultural sources of lignin. Table indicates lignin content of different feedstocks and their geographical distribution around the world (* data collected from [227], *n.a.* data not available)

Lignin source	Composition (%, dry basis)			Global availability of lignin content of the biomass for biorefining* (Mtonnes)	References
	Lignin	Cellulose	Hemicellulose		
Hardwood (alder, beech, maple, oak, teak, etc.)	15–30	38–49	19–40	n.a	[228–230]
Softwood (cedar, juniper, pine, spruce, etc.)	26–34	40–50	7–17	n.a	[229, 231, 232]
Corn stover	12–18	34–36	22–23	Africa: 3.7 America: 25.3 Asia: 15.8 Europe: 4.8	[233, 234]
Rice straw	19–21	35–38	18–25	Africa: 1.8 America: 1.8 Asia: 34.3 Europe: 0.2	[235, 236]
Sugarcane bagasse	20–28	37–42	21–26	Africa: 1.7 America: 18.5 Asia: 12.4 Europe: < 0.0002	[237, 238]
Wheat straw	15–23	30–42	18–29	Africa: 1.9 America: 7.4 Asia: 23.1 Europe: 18.6	[239–241]

**Fig. 1:**
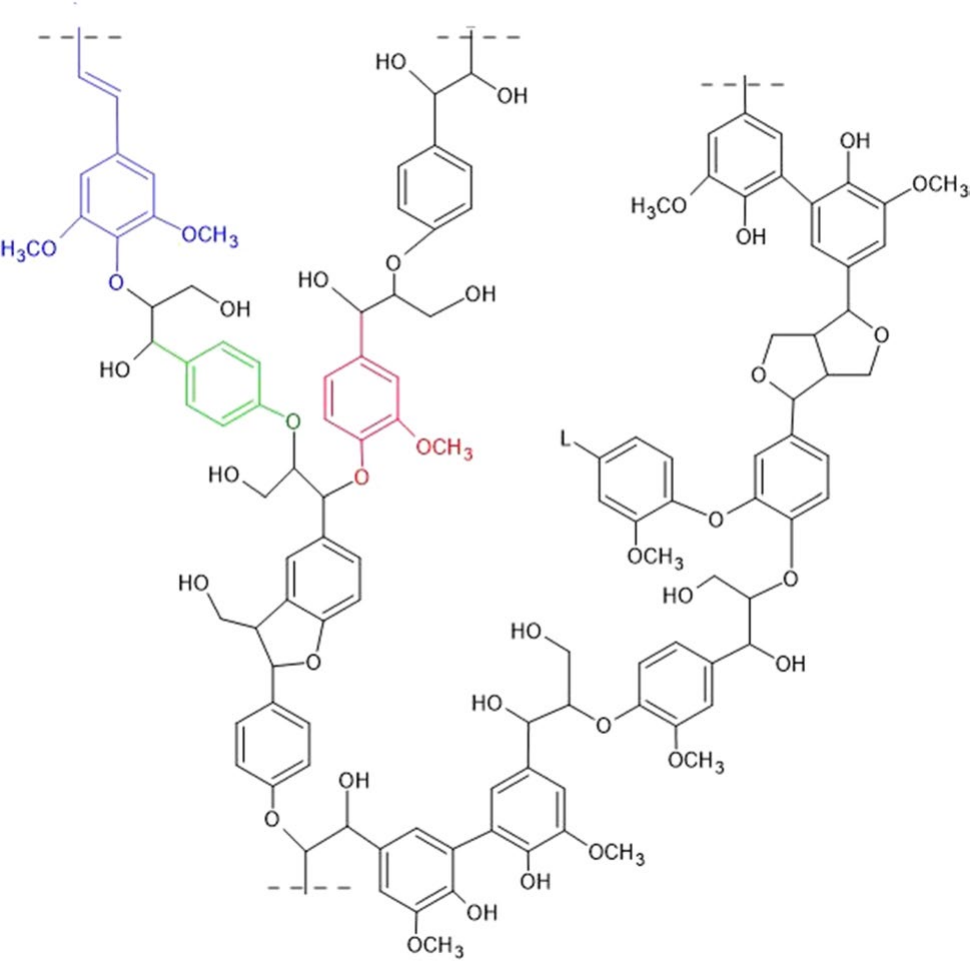
Macromolecular structure of lignin and its major monolignol units. S-units (sinapyl alcohol) are colored in blue, H-units (ρ-coumaryl alcohol) are colored in green, and G-units (guaiacyl alcohol) are colored in red. This structure is adapted from [242]

The chemical composition and lignin content of the biomass depend on the agricultural source and growth conditions. The diversity of the lignin structure leads to the necessity of developing multiple methods to isolate the desired chemical fractions from lignin [28]. Lignin is a high molecular weight complex aromatic heteropolymer mainly composed of three different monomers or monolignol units: (i) p-coumaryl alcohol (H units), (ii) guaiacyl alcohol (G units), and sinapyl alcohol (S units) [29–31] (Fig. 1). The molecular weight of hardwood lignin is lower than that of softwood lignin [32]. Guaiacyl alcohols (G units) constitute approximately 90% of softwood lignin, whereas approximately equal amounts of G and S units appear in hardwood lignin [32]. Since the β-O-4 aryl ether bond is the dominant linkage (approximately 50% of all ether linkages in lignin) in both softwood and hardwood, degradation of this structure is a crucial step in lignin degradation [33, 34].

## Types of lignin and current lignin extraction processes

**Fig. 2:**
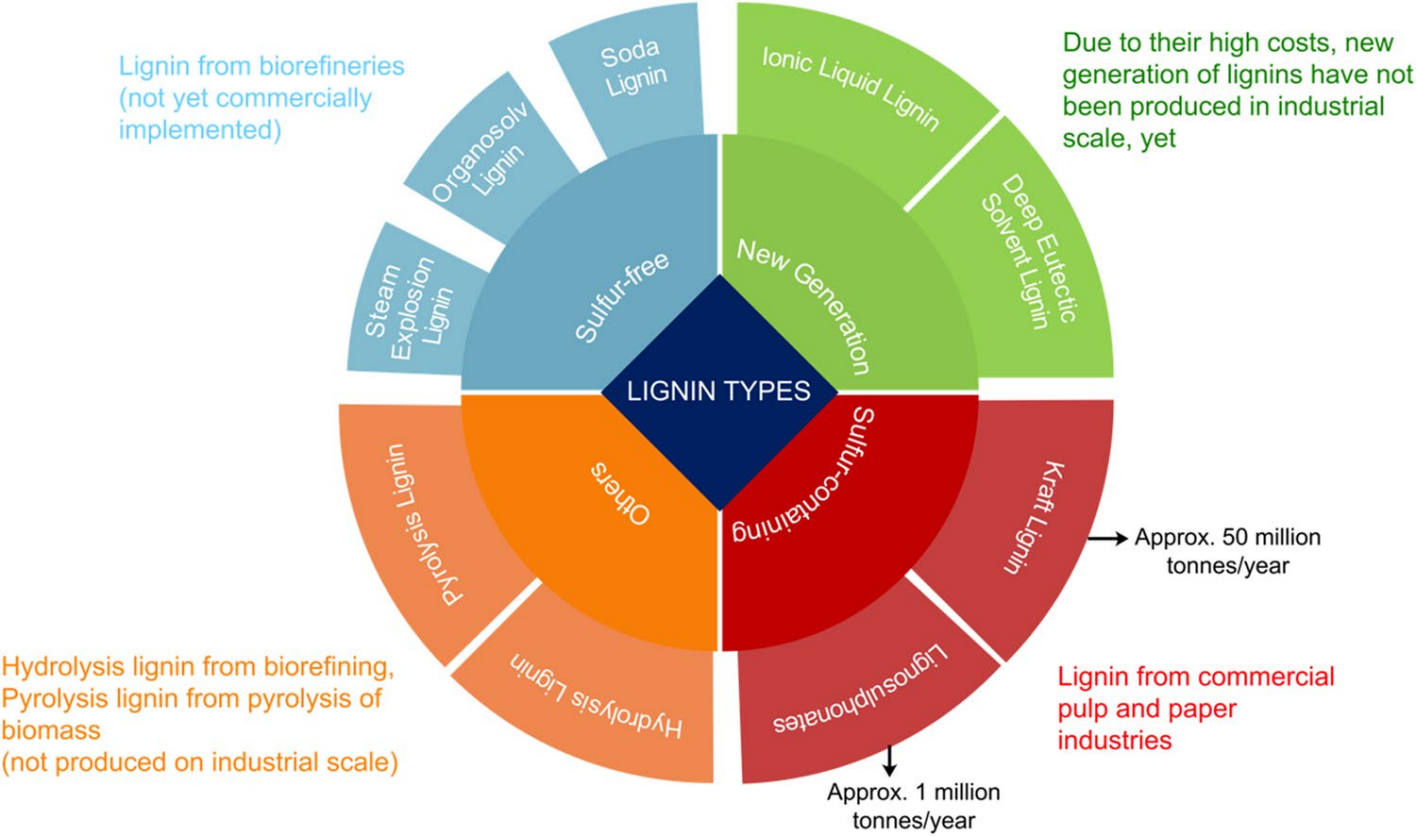
Classification of the most well-known types of lignin. Classification based on source and method of production

The biomass source and the conditions used for extraction and isolation influence the type and purity of the lignin fractions produced. Figure 2 summarizes current lignin types within four major categories as described below.

### Sulfur-free lignins

Soda lignin is a sulfur-free lignin that comes from the soda pulping process. Soda pulping is one of the earliest methods invented for cellulose extraction from lignocellulosic biomass by Watt and Burgess in 1853 [35]. Crop residues such as straw, flax, and bagasse are used in the soda process [36]. In this case, biomass is treated with aqueous NaOH at a temperature between 150 and 170 °C. Due to degradation of lignin and release of carbohydrates during the process, the selectivity of lignin extraction by this method is low [37]. However, some additives/catalysts, such as anthraquinone, can be added to decrease carbohydrate degradation and improve selectivity [38].

In other lignin extraction processes, biomass is treated with a solution containing a polar organic solvent (e.g., alcohols, organic acids, ketones) at high temperature with or without a catalyst [39]. Extraction is based on the solubility of lignin in the organic solvent used [40]. The main advantage of this organosolv method is the easy recovery of the solvent and lignin by distillation and precipitation [41]. The organosolv method is a costly technology, but the quality of the product is considered to be high in that it does not contain sulfites (however it does remains poorly water soluble) [42]. Organosolv lignin contains more phenol hydroxyls and carbonyl groups [43] and hence has high potential for production of phenolic and epoxy resins [44]. The solvents used in an organosolv process are considerably more expensive than the chemicals used in conventional pulping processes [45]. An organosolv method integrated with membrane filtration was evaluated in a previous study, and it was reported that the production cost of the obtained lignin was around 52 €/tonne, which was higher than the cost of Klason lignin (33 €/tonne) [46]. In another study, the economic and environmental impacts of different lignin extraction processes were assessed, and both production costs and potential environmental impacts of organosolv extraction were found to be higher than other methods (Kraft extraction, lignosulfonate extraction, soda extraction, etc.) [42].

In a lignocellulosic biorefinery, removal of lignin can increase the efficiency of enzymatic hydrolysis of the remaining carbohydrate-based polymers which directly affects biofuel production yield. Various chemical, biological, and physicochemical pretreatment methods have been developed to disrupt the recalcitrant structure of lignocellulosic biomass. These methods are not discussed in this review because they focus on degradation of hemicellulose, reduction in cellulose crystallinity, and the amount of carbohydrate extracted rather than on the extraction of lignin. In some cases, however, these methods have been modified to facilitate lignin extraction. Steam explosion is an effective and well-studied physicochemical pretreatment method. In general, hot steam (180–240 °C) is used to break down the structural components of lignocellulosic biomass under high pressure (1.0–3.5 MPa) [47]. In terms of purity and chemical structure, there is a large heterogeneity in the lignin obtained [48]. Compared to Kraft lignin, the lignin fractions released by steam explosion have lower molecular weight and higher solubility in aqueous/organic solvents [49].

### Sulfur-containing lignins

The most well-known type of lignin is Kraft lignin which comes from commercial pulping. In general, Kraft liquor, which contains 16 g of sodium sulfite in 1L of 1 N NaOH solution, is mixed with wood chips in a ratio of 4:1 (w/w, liquid/wood) and cooked at 155–180 °C for 1–2 h [50]. The process temperature and time are dependent on the biomass; most softwoods require higher temperature than hardwoods [51]. Since the pulping process is designed to produce paper and other cellulose products, the produced Kraft lignin has low ß-O-4 content and in most cases does not give good yields of depolymerization products.

The annual global production of Kraft lignin is approximately 45 million tonnes of which 98–99% is incinerated to produce energy and only 1–2% of this material is used for chemical production [52]. Depending on the type of wood and cooking process, the resulting Kraft black liquor is usually composed of 30–45% (w/w) ligneous material, 25–35% (w/w) saccharinic acids, 10% (w/w) formic acid, 3–5% (w/w) sulfur, and 3–5% (w/w) other extractives [53]. The most common method to recover lignin from Kraft liquor is acid precipitation using sulfuric acid [54]. In the Kraft process, the chemical balance between sodium and sulfur is of interest to minimize the production cost. Precipitated lignin can be separated from the liquid via filtration, and the resulting product contains 1.5–3% (w/w) sulfur [41]. CO_2_ acidification and electrochemical acidification processes are emerging alternatives to Kraft pulping. CO_2_ acidification has a lower impact on the sodium-sulfur balance, and the precipitate can be easily filtered [41]. Electrochemical acidification requires lower amounts of chemicals compared to conventional methods, but the low chemical and mechanical stability of ion exchange membranes, membrane fouling, and high operational costs limits their commercial application [41].

Lignosulfonates are produced by sulfurous acid and/or a sulfite salt in the sulfite pulping process. In a traditional lignosulfonate extraction process, wood chips are treated with aqueous sodium sulfite at high temperatures. The cook may have acid, neutral, or alkaline character. In a neutral sulfite treatment process, sulfite liquor contains 15% sodium sulfite and 1.5% sodium carbonate mix with wood chips at 3:1 ratio (w/w, liquid/wood) and cooked at 175 °C for approximately 90 min [50]. Lignosulfonates generally have more sulfur groups than Kraft lignin, and due to the presence of sulfonated groups, they are anionic and water-soluble [55]. This means they may not be easily precipitated by acidifying the liquor [55]. The average molecular weight of hardwood lignosulfonate is 7–11 kDa, while the molecular weight of softwood lignosulfonate is 35–57 kDa [56].

Amine extraction and ultrafiltration are among the most commonly used methods to separate lignosulfonates [57]. The main problems encountered are subsequent removal of amine from the product, the formation of NaCl during re-extraction, foam and emulsion problems, and a time-consuming separation procedure [57]. Consecutive ultrafiltration steps with different molecular weight cut-off (MWCO) membranes are used to separate impurities as well as high molecular weight lignosulfonates [56]. Membrane-based filtration holds great promise for an economical and environmentally sustainable recovery of highly pure lignin [58]. Another strategy to recover high-purity lignosulfonates is including a fermentation step to utilize residual sugars before membrane filtration [41].

Around 66% of lignosulfonate is currently used as fuel for energy production during the pulping process [59]. The annual global production of lignosulfonates is growing and estimated to reach 1.75 M tonnes by 2025 [60]. Kraft lignin is the second most commonly used lignin after lignosulfonates [41]. The total annual global production of technical lignin is approximately 1.65 Mt, among which lignosulfonates dominate 80% of the market [61]. Lignosulfonates are commonly used for commodities like coal briquettes, ceramics, plywood production, and water-reducing concrete additives [37, 62]. Diverting a proportion of lignin from low-value products to high-value chemicals would greatly improve overall process economics.

### Next-generation “greener” lignins

Ionic liquids are becoming attractive green alternatives to conventional organic solvents [63]. They can dissolve high amounts of biomass and can be designed to be multifunctional solvents [64]. Various types of ionic liquids have been used as a pretreatment method to fractionate lignocellulosic biomass and enhance cellulose digestibility [63, 65–67]. Ionic liquids can dissolve up to 20% (w/w) lignin, and the solubility is correlated to the structure of the cation and anion, particularly the anion [68]. Protonic ionic liquids that are produced via an acid–base neutralization process are relatively cost competitive and demonstrate high lignin–extraction efficiency [69]. The solubility of different types of lignin in various ionic liquids has recently been reviewed, and it was shown that ionic liquids possessing aromatic cations have high lignin solubility, while those with nonaromatic cations have lower solubility [70]. However, the lignin dissolution mechanism is not fully understood, and further evaluation is needed to understand the properties and potential of the extracted lignin [71].

Deep eutectic solvents are another alternative to ionic liquids [72]. They are non-flammable, non-toxic, and sometimes biodegradable. A deep eutectic solvent is a liquid mixture formed by hydrogen-bond donors and acceptors [73]. Generally, the final melting point of the mixture is much lower than the individual components, usually < 100 °C [74]. Unlike ionic liquids, deep eutectic solvents can be easily prepared from available materials, and their cost is lower than that of ionic liquids [75]. The lignin extraction yield of various deep eutectic solvents for various lignocellulosic feedstocks and pretreatment conditions have also been reviewed. Compared to other deep eutectic solvents ChCl/oxalic acid and ChCl/lactic acid-based solvents had the best delignification yields [70]. In a recent study, these solvents were evaluated for lignin separation from rice straw. The results showed that nearly 60% of the lignin in the biomass can be separated at a high purity (> 90%) [74]. In another study, five different deep eutectic solvents were used for preferential dissolution of lignin in loblolly pine at 60 °C, and depending on the solvent type, various lignin solubilities (9–14%, w/w) were reported [75]. This means that deep eutectic solvents can be employed to facilitate selective lignin extraction from lignocellulosic biomass [76]. Their efficacy is significantly dependent on the chemical composition of the biomass, and recent developments in this area have been critically reviewed in another study [77]. However, to date there are very few studies on the characterization of deep eutectic lignin and its potential in pharmaceutical applications. The cost and scale of production of both ionic liquids and deep eutectic solvents also need to be addressed.

### Other lignins

Finally, other types of lignin can be separated from lignocellulosic biomass that undergoes different pretreatment processes. Pyrolysis lignin and hydrolysis lignin are typical examples. Pyrolysis is a thermal decomposition process that converts lignocellulosic biomass into a solid residue (bio-char), bio-oil, and gas under a non-oxidizing inert atmosphere at high temperatures (> 400 °C). Depending on the biomass and operating conditions used, a wide range of lignin-derived materials can be found in pyrolysis products. Recently, a range of lignin-derived compounds, such as guaiacol and 2-methoxy-4-vinylphenol, were quantified by thermogravimetric analysis of products from pyrolysis of pine, bamboo, corncob, and corn stover [78]. In general, bio-oils contain a significantly less water and acetic acid and larger amounts of sugars and other products generated during lignin pyrolysis [79]. Pyrolytic lignin can be separated from bio-oil by pouring the bio-oil in iced water under stirring [80]. Previously, pyrolytic lignin obtained from pyrolysis of hardwood biomass was evaluated for bio-composite production and compared with soda and Kraft lignins. The study showed that pyrolytic lignin has potential for resin and adhesive applications, but Kraft lignins were preferred for application in bio-composites based on polyethylene [80]. More studies are needed however to determine the real industrial potential of pyrolytic lignin.

Acid saccharification of lignocellulosic biomass and woody materials has been developed to facilitate bioethanol production [81]. Hydrolysis lignin is the term that describes the left-over lignin that is precipitated from acid hydrolysis black liquor. Depending on the composition of the biomass and the process conditions, high quantities of hydrolysis lignin can be produced. For example, in a coniferous wood to ethanol plant, 0.9 tonne of coniferous wood yields 160–175 kg of ethanol and produces 350–400 kg of lignin as by-product [82]. Therefore, finding effective and economic processes to valorize this by-product is a major focus [83–85]. Recently, lignin generated as a by-product of acid hydrolysis of white poplar was characterized and compared with lignin generated from Kraft pulping and organosolv hydrolysis of the same biomass [86]. Results showed that hydrolysis lignin exhibited extensive depolymerization and lower molar mass and higher phenolic content. Furthermore, results showed that hydrolysis lignin has high thermal stability and for this reason could be used for developing flame-retardant materials. However, acid treatment eliminates the α-hydroxy group in ß-O-4 units, leading to highly condensed structures with low ß-O-4 content which makes this lignin difficult to depolymerize.

### Accessing lignin-derived chemical building blocks

In literature, there are multiple studies showing the pharmaceutical potential of various compounds derived from lignin. To produce these from lignin, several pretreatment processes including pyrolysis, chemical oxidation and hydrolysis, and microbial and enzymatic treatment methods have been carried out [85, 87–90]. The oxidative cleavage of C–O–C and C–C bonds in lignin can result in monomeric phenols together with CO, CO_2_, and H_2_O. However, oxidative methods become undesirable due to the production of free radicals as a side product [91]. Solid catalysts can also be used for selective cleavage of C-O ether bonds in lignin. In [92] a solid catalyst (SiO_2_-Al_2_O_3_) was used under an inert atmosphere to depolymerize lignin into aromatic monomers with high yields (60%) at 250 °C. In another study, a novel catalyst, Ni/CaO-H-ZSM-_5_(60), has showed promising performance for C-O bond cleavage under very mild conditions (141 °C, 1 MPa H_2_), and the C-O bond of β-O-4 has been completely cleaved within 60 min [93].

Even though white-rot basidiomycete fungi has been extensively studied for biological lignin degradation [94, 95], in nature, there are some bacteria capable of deconstructing lignin [96]. Furthermore, some bacteria have metabolic pathways that simultaneously convert lignin into high-value products such as ferulic acid and vanillin. Since 2010, there has been a resurgence of interest in lignin-oxidizing enzymes from soil bacteria [89]. Recently, *Paraburkholderia aromaticivorans* AR20-38, a bacterium isolated from Alpine forest soil, was used to convert ferulic acid (5–10 mM) to vanillic acid at low and moderate temperatures (10–30 °C), and the strain showed high bioconversion yield (85–89%) without inhibition of growth [97].

A more recent strategy is the use of lignin-degrading enzymes to imitate the lignin degradation process in nature [98]. Microbial β-etherases catalyzing the reductive cleavage of β-O-4 bonds are promising candidates for biotechnological lignin degradation [99]. Microbial lignin degrading enzymes including fungal peroxidases and bacterial stereoselective enzymes have been described elsewhere [100]. Enzymatic methods can be combined with steam explosion to extract ferulic acid from wheat bran [101]. This study showed that Alcalase and Termamyl pretreatment increased the ferulic acid extraction yield by up to 20-fold (0.82–1.05 g/kg bran).

Potential therapeutic applications of some of the major lignin-derived phenolic compounds are discussed in the following sections.

## Opportunities and challenges for the production of lignin-based chemicals and pharmaceuticals

**Fig. 3:**
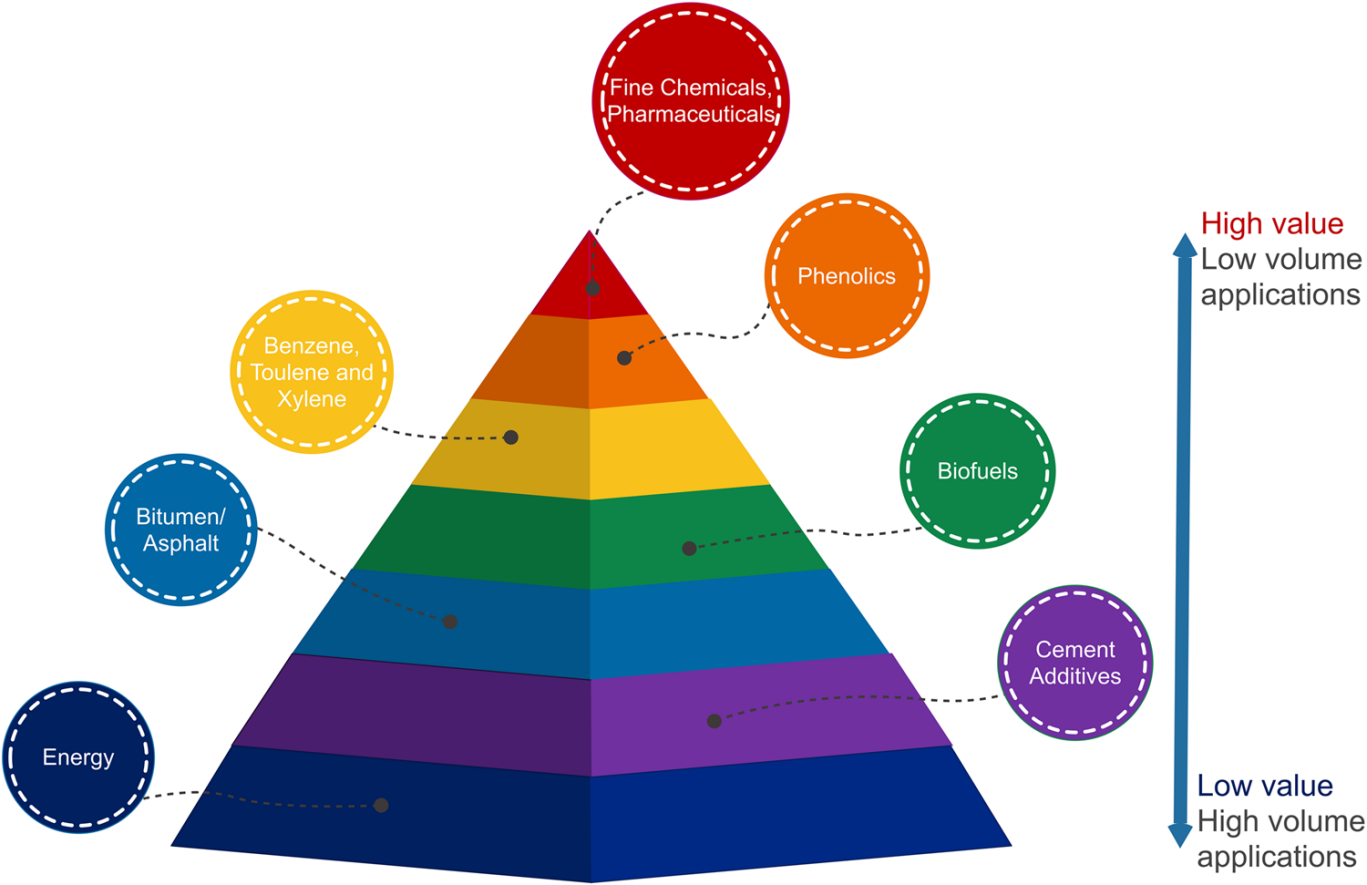
Potential lignin applications and relative values of different product classes. Adapted from [243]

The sustainability and economic viability of biorefineries and paper mills depend upon utilization and, ideally, valorisation of all three of the major biopolymers present in lignocellulosic biomass. Extracting specific chemicals from lignin remains challenging and resource intensive, as described in Section 3, and while many different products can be isolated, only a few are considered promising for industrial scale production [102]. Figure 3, the value pyramid, categorizes some of the most important products obtainable and their relative commercial value. Products with pharmaceutical applications are clearly top of the pyramid and offer a way to improve biorefinery economics. Such applications are therefore of commercial interest provided they do not interfere with primary material flows (e.g., cellulose utilization for bioethanol production) or key existing operations (e.g., anaerobic digestion for waste treatment).

A number of approaches have thus been developed to convert lignin into pharmaceutical products. From an engineering perspective, extracting the desired compounds from biomass-derived lignin is challenging due to its complex structure (Fig. 1) and the frequently low concentrations of the target compounds (typically < 10%). Furthermore, most thermo-chemical processes for lignin fractionation causes structural modifications, while more selective enzymatic treatments are expensive [103].

Generation of complex mixtures by traditional lignin degradation methods is one of the biggest challenges of lignin-based chemical and pharmaceutical production. Recent developments in the discovery of bacterial enzymes for lignin degradation and their potential for generation of renewable chemicals were described elsewhere [89]. However, strategies like non-catalytic or catalytic thermochemical transformation [104], chemo-catalytic and biocatalytic conversion of lignin [105] that enable the simultaneous release of a number of pharmaceutical precursors, with high selectively and yield, while maintaining the existing lower-value application of the residual lignin would appear the most attractive.

For biorefinery operations, specific challenges are ensuring that lignin utilization does not jeopardize carbohydrate extraction or increase the capital cost [106] and that the depolymerization method used does not degrade the extracted compounds due to their low starting concentrations [107–109]. Improved methods for the chemical, physical, and structural characterization of lignin, and extracted fractions, are needed that can also be used for biorefinery monitoring and control [41, 103, 108].

Reactive oxygen species (ROS) which include hydrogen peroxide (H_2_O_2_), hypochloride (OCl^−^), hydroxyl radical (•OH), and superoxide (O_2_•^−^) are unstable molecules that react with other molecules in a cell. They are commonly generated, transformed, and consumed by living cells during normal metabolism. They function in cells as signalling molecules, and maintaining a basal level of ROS in cell is essential for life [110]. When ROS production increases, they damage vital cellular structures, such as DNA, proteins, and lipids. Antioxidants are chemicals that can inhibit or slowdown the damages caused by ROS by directly reacting with free radicals or indirectly inhibiting the activity enzymes that generate free radicals or enhancing the intracellular activity of antioxidant enzymes [111, 112]. Phenolic hydroxyl groups are the main reason of antioxidant and antimicrobial properties of lignin and lignin extracts [113]. In general, it is considered that the antioxidant effects of lignin are derived from the scavenging activity of phenolic structures on oxygen containing ROSs [114]. The influence lignin extraction methods have on the antimicrobial activities of various lignin-based materials was discussed in a previous study [115]. For example, Wang et al. used a successive ethanol–water dissolution method to fractionate enzymatic hydrolysis lignin (EHL) and reported that successive fractionation was an effective way to enhance the antimicrobial activity of EHL extracts on Gram-positive (*Staphylococcus aureus* and *Bacillus subtilis*) and Gram-negative (*Escherichia coli* and *Salmonella enterica*) bacteria [116]. They concluded that the better solubility and higher hydroxyl content of the lignin fractions might be responsible for this improvement.

**Fig. 4:**
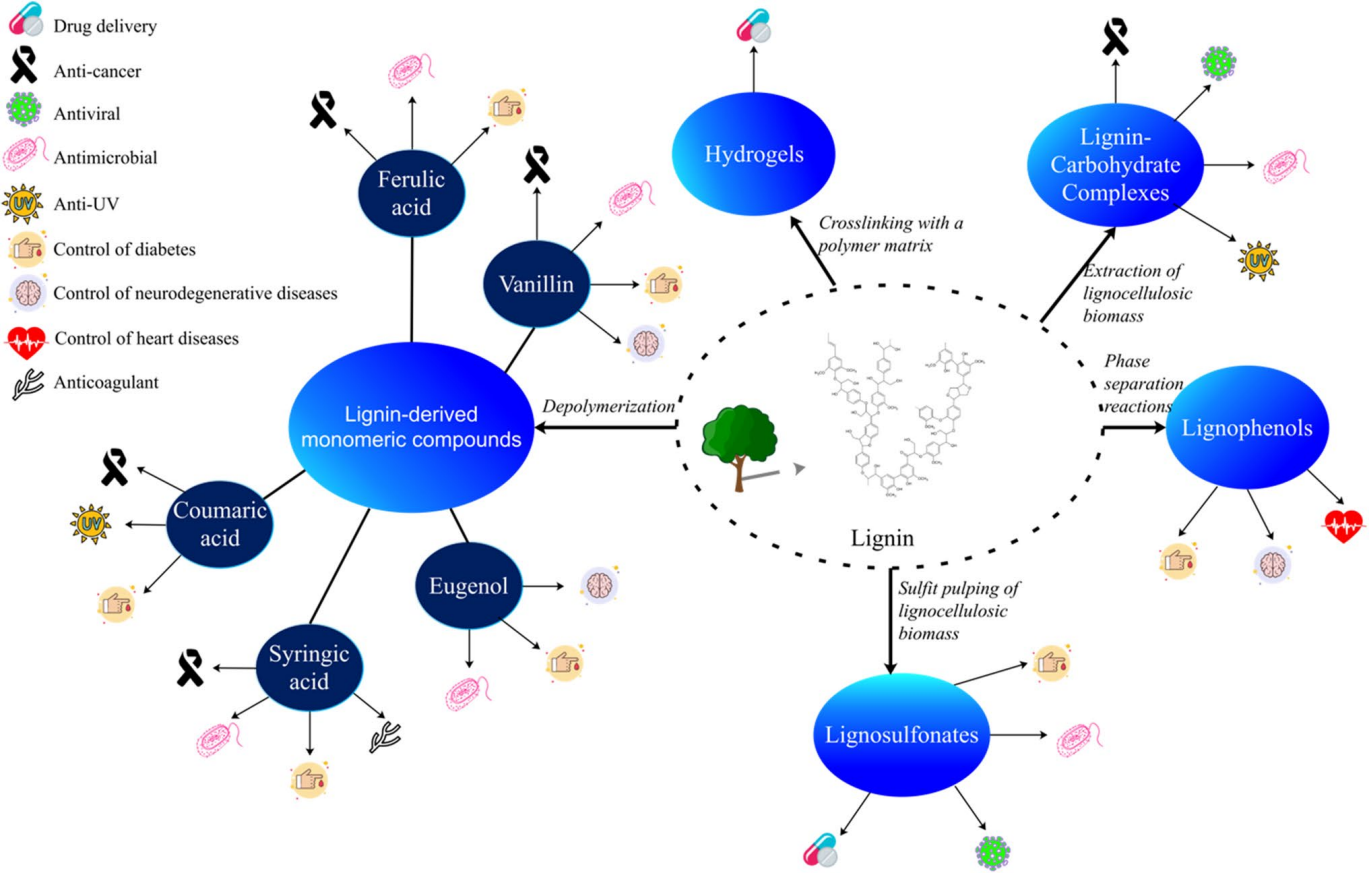
Overview of potential therapeutic applications of lignin-derived feedstocks. The major potential of lignin-based monomeric compounds is their anticancer and antidiabetic use. Lignin-carbohydrate complexes (LCC) also show important anticancer potential. Unlike the other lignin-based compounds, lignophenols have unique potential for control of heart disease

Figure 4 summarizes the main lignin derivatives exploited to date and their potential therapeutic application as described in literature. Even though these studies show the potential for pharmaceutical and therapeutic applications, these approaches have not yet been commercialized. Furthermore, most of the examples have relied on use of chemical grade compounds for clinical trials rather than biomass-derived products. The following sections describe the relationship between lignin-derived compounds and their potential therapeutic applications.

## Pharmaceutical and healthcare applications of lignin-derived compounds and complexes

### Individual compounds derived from lignin

#### Vanillin and vanillic acid

**Fig. 5:**
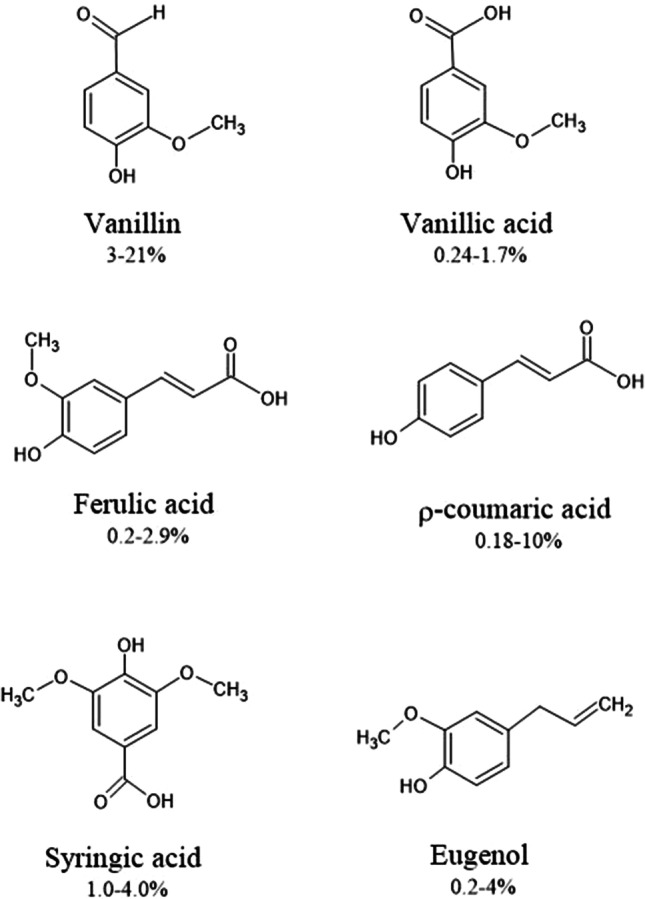
Major monomeric lignin derivatives with potential pharmaceutical application. Figure also indicates extraction yields of each derivative (% w/w, based on lignin content) isolated from different lignin sources [164, 244–251]

The structures of the major lignin-derived monomeric phenolic compounds are provided in Fig. 5. Vanillin (4-hydroxy-3-methoxybenzaldehyde) is usually present at the highest concentrations (~ 20%) and is currently the only phenolic compound manufactured on an industrial scale from biomass, mainly via the Kraft process [117]. It is produced on a scale of more than 9000 tonnes per year [118]. Only 5% of global vanilla production comes from the pod of *Vanilla orchid*, while 95% of vanillin is produced synthetically, and 15% of synthetically produced vanillin is derived from lignin [117]. Various strategies have been developed to produce vanillin from Kraft lignin [119, 120] and ferulic acid [121, 122]. Vanillin, the major flavor constituent of vanilla, has a wide range of applications in the food and beverage industry, perfumery, and the synthesis of several pharmaceutical chemicals [123]. Trimethoprim and L-DOPA (L-3,4-dihydroxyphenylalanine) are the most well-known pharmaceuticals that can be produced from vanillin. Trimethoprim is an antibiotic used in the treatment of urinary infections, and L-DOPA is used to treat Parkinson’s disease, as it is a precursor for the neurotransmitter dopamine [124]. The protective effect of vanillin on diabetic nephropathy, a common complication of diabetes which leads to renal dysfunction, was also studied [125]. Experiments conducted on diabetic rats showed that vanillin significantly decreased the fasting blood glucose level, and vanillin administration at a dose of 100 mg/kg improved kidney function. The authors concluded that treatment with vanillin exhibited a potent reno-protective action against diabetic nephropathy, and vanillin administration in the early stages of diabetic nephropathy should be a focus of future human-based clinical studies.

In the literature, there are also some studies that investigated the anticancer activity of vanillin and vanillin-based chemicals. The in vivo anticancer activity of vanillin semicarbazone against Ehrlich ascites carcinoma cells in Swiss albino mice was previously studied [126]. The study showed that vanillin semicarbazone at a dose of 10 mg/kg increased the life span to more than 88.9%; hence, it can be considered a potent anticancer agent [126]. Elsherbiny et al. investigated the synergistic effect between vanillin and doxorubicin which is an anthracycline antibiotic widely used as a chemotherapeutic agent for breast cancer [127]. The study showed that vanillin (100 mg/kg) provided protection from the toxic side effects of doxorubicin and that the mean survival time of the mice treated with the combination of these two chemicals (88.3 days) was significantly higher than the group treated with only doxorubicin (33.7 days). Vanillic acid (4-hydroxy-3-methoxybenzoic acid) is an oxidized form of vanillin, and it is also used as a flavoring agent. Similar to vanillin, the effects of vanillic acid on the toxic effects of cisplatin, another extensively used cancer drug, was investigated using an animal model [128]. This study showed that male albino rats treated with vanillic acid (50–100 mg/kg) restored the elevated levels of renal functions and reduced the antioxidant status to near normalcy when compared to animal group treated only with cisplatin. These results demonstrate that vanillin and vanillic acid can be used as a combinatorial regimen in cancer therapy.

#### Ferulic acid

Ferulic acid (4-hydroxy-3-methoxycinnamic acid) is a lignin-based phenolic acid that can be used to produce vanillin and vanillic acid. In the plant cell wall, ferulic acid is usually found cross-linked with hemicelluloses via ester bonds [129]. It can be obtained by hot water [130], deep eutectic solvents [131], or alkaline processes [132], and it has been used in traditional Chinese medicine for treatment of cardiovascular and cerebrovascular diseases [133]. As a natural antioxidant, it exhibits an ability to scavenge free radicals, and it presents a wide array of antioxidant, antimicrobial, anti-inflammatory, antidiabetic, and anti-carcinogenic activities [129, 134]. Curcumin, a dimer of ferulic acid, also targets critical genes associated with angiogenesis, apoptosis, cell cycle, and metastasis, hence it has been considered an anticancer agent [135]. Lin et al. investigated the activity of ferulic acid on human keratinocyte HaCaT cells treated by UVB radiation [136]. The study showed that ferulic acid could inhibit UVB-induced carcinogenesis and has potential anti-carcinogenic properties on UVB-induced epidermic tumor development. In another study, the radiosensitizing effect (increasing the lethal effects of radiation) of ferulic acid was tested in two cervical cancer cell lines (HeLa and ME-180) [137]. The authors reported enhanced oxidative DNA damage and apoptotic morphological changes in ferulic acid and radiation-treated cells. They found that ferulic acid enhances the lethal effects of radiation and decreases the cell viability and survival rate. Fahrioğlu et al. examined the effects of ferulic acid on gene expression, viability, colony formation, and mitigation/invasion in cultured MIA PaCa-2 human pancreatic cancer cells [138]. They reported that ferulic acid behaves as an anticancer agent by affecting cell cycle, apoptotic, invasion, and colony formation of cancer cells. Zhang et al. evaluated the antitumor activity of ferulic acid in the breast cancer cell line MDA-MB-231 [139]. They found that ferulic acid treatment decreased viability and increased apoptosis and suppression of metastatic potential in breast cancer cells. These results suggest that ferulic acid might be an effective therapeutic agent against breast cancer and has potential as an effective treatment for different types of cancer.

Furthermore, there are a small number of studies investigating the effects of ferulic acid and its combinations with other antioxidants in diabetes. Song et al. tested the activity of ferulic acid on obese, diabetic rats [140]. They found that it significantly increased the antioxidant activity in the plasma, heart, and the liver. They also reported activity against oxidative stress in obese rats with late-stage diabetes. In another study, the protective effects of ferulic acid on protein glycation, lipid peroxidation, membrane ion pump activity, and phosphatidylserine exposure in high glucose-exposed human erythrocytes were investigated [141]. The authors reported that ferulic acid (10–100 µM) significantly reduced the levels of glycated hemoglobin (HbA1c), and at 0.1–100 µM, it inhibited the lipid peroxidase in erythrocytes which was associated with increased glucose consumption. These results show that ferulic acid is capable of improving the effects of hyperglycemia and preventing vascular dysfunction associated with diabetes.

#### Coumaric acid

Coumaric acid is a hydroxy derivative of cinnamic acid, and in nature, its most commonly available form is ρ-coumaric acid [142]. It can be produced by alkaline hydrolysis [143] and attenuates UV-induced cytotoxicity making it an active ingredient in cosmetics [144]. Similar to ferulic acid, coumaric acid is a well-known plant-based antioxidant.

In several cases, its antioxidant activity was tested together with other phenolic compounds such as ferulic acid and caffeic acids. Yeh et al. studied the lipid-lowering and antioxidative activities of ρ-coumaric acid, ferulic acid, and caffeic acid [145]. They reported that these phenolic compounds significantly lowered the plasma lipid and hepatic cholesterol levels and enhanced antioxidant capacity in high cholesterol-fed rats. In another study, the effects of ρ-coumaric acid against the hippocampal neurodegeneration in type 2 diabetic rats were investigated [146]. This study showed that ρ-coumaric acid may inhibit hippocampal neurodegeneration via its potent antioxidant, anti-inflammatory, and anti-apoptotic properties. Therefore, the authors recommended this compound as a promising adjuvant agent against brain neurodegeneration in diabetics.

Antidiabetic and antihyperlipidemic activities of ρ-coumaric acid in rats were evaluated in a previous study [147]. This study revealed that ρ-coumaric acid protects pancreatic β-cells to control hyperglycemic excursions and improves metabolic disorders via GLUT 2 activation. Recently, antidiabetic activities of 11 phenolic acids, including ρ-coumaric acid, were compared with metformin, a well-known medication for the treatment of type 2 diabetes [148]. Results showed that all phenolic acids had equivalent or more potent effects on glucose uptake in HepG2. This study also reported that coumaric acid is among the top three phenolic acids that showed the highest glucosidase inhibitory activity.

In the literature, there are some studies that have investigated the anticancer potential of ρ-coumaric acid. Protective effects of ρ-coumaric acid and ferulic acid against colon cancer were studied on endothelial tumor cell line Caco-2 [149]. The study reported that both of these chemicals showed anti-proliferative effects on Caco-2 human cancer cells and decreased the number of cancer cells to 43–75% of the control after 2–3 days of treatment. Roy et al. investigated the activity of ρ-coumaric acid and ferulic acid on human colorectal cancer cell line HCT 15 and epidermal growth factor receptor (EGFR) that may have an important role on provoking colorectal cancer [150]. They found that these chemicals were able to inhibit EGFR at its active site. Furthermore, the cytotoxicity experiments showed that both ρ-coumaric acid and ferulic acid were efficient in killing colorectal cancer cells.

#### Syringic acid

Syringic acid (4-hydroxy-3, 5-dimethoxybenzoic acid) is another phenolic known for its strong antioxidant activity and can be obtain via alkaline hydrolysis [151]. It can be used as a therapeutic agent in various diseases such as diabetes, cancer, and liver damage [152]. It can modulate the dynamics of several biological targets such as transcriptional and growth factors [152]. The leaves and bark of different *Quercus* species (a small oak tree) have been used to extract syringic acid and other phenolic compounds for assessment of their biological activities [153]. *Quercus infectoria* is one of the most popular traditional medicines in Asia and used to treat wound infections and toothache [154]. In 1979, syringic acid (extracted from powdered galls of *Quercus infectoria* using solvent extraction) and the neuropharmacological activity of a syringic acid-rich extract were tested on mice [155]. The effect of syringic acid against thrombogenesis and platelet activation was investigated on male Sprague–Dawley rats [156]. They found that syringic acid attenuated the development of thrombosis and thromboembolism by inhibiting clot formation and procoagulant protease activity. These findings demonstrated that syringic acid has significant potential as an antithrombotic and antiplatelet agent that can be used against cardiovascular disease and its possible complications.

The antimicrobial activity of syringic acid and syringic acid containing plant extracts was also assessed against different bacteria and fungi [157–159]. Shi et al. reported that syringic acid retarded bacterial growth of *Cronobacter sakazakii*, an opportunistic pathogen that has been implicated in bacteraemia and neonatal meningitis, and caused cell membrane dysfunction. They also indicated that it has a strong potential to develop natural preservatives to prevent *C*. *sakazakii*-related infections. Abaza et al. investigated the antimitogenic and chemo-sensitizing activities of syringic acid isolated from *Tamarix aucheriana* (salt cedar plant) against human colorectal cancer cell lines SW1116 and SW837 [160]. They reported that syringic acid showed a time and dose-dependent antimitogenic effect against cancer cells with little cytotoxicity on normal fibroblasts. They also reported that it sensitized cancer cells to standard chemotherapies and increased their sensitivity up to 20,000-fold compared to standard drugs.

#### Eugenol

Eugenol (4-allyl-2-methoxyphenol) is another chemical derived from lignin in woody biomass. Eugenol can be converted to ferulic acid and vanillin via different biochemical pathways [161–163]. It can also be generated by the depolymerization of lignin along with variety of active biomaterials. Varanasi et al. reported that the production of lignin-based chemicals such as eugenol, phenols, guaiacols, syringols, and catechols are dependent on the starting biomass concentration and dissolution temperature; they produced approx. 2 g eugenol from 1 kg of low sulfonate alkali lignin after dissolution at 160 °C for 6 h at 3% biomass loading [164].

Eugenol is generally used as a flavoring agent and food additive. In the literature, there are also some studies on its antioxidant and antimicrobial properties. Zhang et al. tested the activities of eugenol and isoeugenol against several foodborne pathogens such as *S. aureus*, *Bacillus subtilis*, *Listeria monocytogenes*, *E. coli*, *Salmonella typhimurium*, and *Shigella dysenteriae* [165]. They showed that both of the compounds had strong antioxidant and antimicrobial activities. Furthermore, they exhibited protective effects against DNA damage. In another study, eugenol-bearing oxypropanolamine derivatives were developed, and their antimicrobial activities were tested against various multidrug-resistant bacteria including *Acinetobacter baumannii*, *Pseudomonas aeruginosa*, *E. coli*, and *S. aureus* [166]. These derivatives showed strong antibacterial effects on bacterial strains and inhibited some important metabolic enzymes like α-glycosidase (involved in the digestion of carbohydrates), cytosolic carbonic anhydrase I and II (its I form is a key enzyme in aqueous humor production in eye, and its II form is found in renal tubes, brain, and osteoclasts), and acetylcholinesterase (performs a key role in the functioning of cholinergic neuronal pathways). Hence, the authors concluded that these chemicals can be investigated further for the treatment of some important diseases such as epilepsy, ulcers, glaucoma, osteoporosis, and neurological disturbance. The protective effects of eugenol as a therapeutic intervention agent under diabetic condition was studied in vitro and in vivo animal-based models [167]. One study showed that eugenol exposure rescued SHSY5Y cells from glucose-induced death and increased cell survivability. In the animal-based model, eugenol treatment significantly lowered the mean body weight and blood glucose levels of diabetic rats. In another animal-based study, the antidiabetic effects of eugenol were demonstrated by significant reduction levels of serum glucose, triglyceride, and cholesterol in diabetic male adult Sprague–Dawley rats [168]. Moreover, this study showed that eugenol treatment (10–20 mg/kg) facilitated insulin sensitivity, and it could be a promising therapeutic agent to prevent type 2 diabetes.

### Lignin-derived fractions and complexes

#### Lignin fractions obtained by solvent extraction

Solvent fractionation is a convenient method to fractionate technical lignins or depolymerized lignins, which generates a set of fractions of varying molecular weight and hydrophobicity useful for different applications. These fractions have been reported to show enhanced antioxidant activity, antibacterial activity, and UV absorption properties, the latter useful for sunscreen applications [169, 170]. These fractions can be employed as complex mixtures or can be further purified if a single active species can be identified.

#### Lignophenols

Lignophenol, a lignin-based functional polymer, can be separated from lignin through phase separation reactions composed of phenol derivatives and concentrated acid [171]. Even though lignophenols have high antioxidant properties, their physiological role and medical potential have not been well characterized [25, 172]. In the literature, some in vitro and animal-based studies have shown the pharmaceutical potential of lignophenols. In one study on streptozotocin-induced diabetic rats, lignophenols were able to attenuate oxidative and inflammatory damage in the kidney by suppressing excess oxidative stress and the infiltration and activation of macrophages in the diabetic kidney [173]. In another study, lignophenols played an important role in improvement in the vascular impairment of diabetes by reducing the vascular oxidative stress and inflammation via inhibition of NAD(P)H oxidase [174]. These results indicate that lignophenols have potential for control of the most common diseases of the twenty-first century: diabetes and obesity. Furthermore, lignophenol derived from bamboo lignin using a phase-separation technique has showed potent neuroprotective activity against H_2_O_2_-induced apoptosis in human neuroblastoma cells (SH-SY5Y) by preventing caspase-3 activation [175, 176]. This suggests that lignophenols are also promising neuroprotectors which can be used to delay the progress of neurodegenerative diseases. In another study, it was reported that lignophenols can decrease secretion of oleate‐induced apolipoprotein‐B, a lipoprotein which is positively correlated with the incidence of coronary heart disease and atherosclerosis, in HepG2 cells (human hepatocellular carcinoma cell line) [172].

#### Lignosulfonic acid

As described in Section 3.2, water-soluble lignosulfonates (salts of lignosulfonic acid) are the major by-product of the sulfite pulping process. They have been shown to be a high-value raw material for fine chemicals such as vanillin [177]. Lignosulfonic acid (LSA), a low-cost lignin-based polyanionic macromolecule, is generated as a by-product from the pulp and paper industry.

The known antiviral and antimicrobial activity of LSA highlights its potential as a low-cost pharmaceutical agent. Gordts et al. investigated the anti-HIV and anti-HSV activity of pure LSA (commercial product) in various cellular assays [178]. They demonstrated that HIV and HSV infection in T cells was blocked by LSA and that LSA has strong inhibitory activity on HIV replication. They also indicated that LSA targeted the envelope proteins and did not show antiviral activity against non-enveloped viruses.

In the literature, there are also some studies investigating the potential of LSA for controlled drug release. LSA and gelatine blend microspheres were developed by cross-linking with glutaraldehyde and used for controlled release of an anti-malarial drug [179]. This study indicated that microspheres enhanced the release rates of the drug up to 10 h and the drug release profiles were pH-sensitive. In a similar study, LSA and sodium alginate blend microspheres were used to develop a polymer matrix for controlled release of an antibiotic (ciprofloxacin) [180]. Based on the results, the developed carrier was found to be suitable for controlled drug delivery for gastrointestinal applications.

Hasegawa et al. studied the inhibition effects of LSA on intestinal glucose absorption [181]. They found that, in human colorectal adenocarcinoma cells, LSA inhibited 2-deoxyglucose uptake and during their in vivo rat experiments found that LSA significantly suppressed the rise in blood glucose levels through inhibition of α-glucosidase activity and intestinal glucose absorption. Similarly, due to the inhibition of α-glucose activity and intestinal glucose absorption, feeding diabetic KK-Ay mice with LSA significantly suppressed the increase of the serum glucose levels [182]. These results suggest that as well as lignophenols, LSA might be used to control obesity and diabetes.

#### Lignin carbohydrate complexes

Some polysaccharides in the cell wall of lignified plants are covalently linked to lignin to form lignin-carbohydrate complexes (LCCs) [183]. There are eight different types of lignin-carbohydrate bonds: benzyl ether, benzyl ester, glycosidic, phenyl glycosidic, hemiacetal linkages, acetal linkages, ferulate ester, and diferulate ester bonds [184]. Phenyl glycoside, benzyl ethers, and ester linkages are the main three types of LLC linkages in wood [185], whereas ferulate and diferulate esters are the prevalent LCC linkages in non-wood plants [184]. Benzyl ethers and phenyl glycoside linkages in wood can be easily cleaved under acidic conditions [185]. Due to the presence of different types of lignin and polysaccharides in different lignocellulosic biomasses, the composition and the structure of natural LCCs is very diverse [184]. The presence of LCC, that is either naturally formed or generated during processing, is considered to be one of the reasons for difficulties in chemical and biological processing of lignocellulosic biomass [186]. Recently, six types of LCC fractions were extracted from Eucalyptus by aqueous dioxane and then precipitated sequentially by 70% ethanol, 100% ethanol, and acidic water [187]. This study showed that the low molecular weight LCC containing a high amount of carbohydrate (60–63%) was precipitated by the first extraction step (70% ethanol). In a recently published study, glucomannan-lignin and glucuronoxylan-lignin were found to be the main structures in the LCCs extracted from hot water pretreatment liquor of poplar [188]. The authors also reported that the glucuronoxylan-lignin complex can be enriched by increasing the process temperature and LLCs extracted from poplar could be suitably modified by changing the temperature.

There are several reports on LCCs possessing antiviral effects against Herpes simplex (HSV) (types 1 and 2), HIV-1, and influenza viruses [189–192]. A pilot study with 48 healthy patients, with active lesions of HSV-1, was undertaken to evaluate anti-HSV-1 activity of LCC obtained from pine cones via an alkaline process. The study showed that the majority of the patients reported a reduction in symptoms after the LCC-ascorbic acid treatment, suggesting its possible applicability for the treatment of HSV-1 infection [193, 194]. Previously, LCCs obtained from cocoa mass and cocoa husk by 1% NaOH extraction and acid precipitation process have shown anti-HIV activity [195]. The study indicated that LCCs stimulated the growth of human MT-4 cells by inducing hormesis and that the cocoa mass LCC has higher anti-HIV activity than that of cocoa husk LCC. In another study, LCCs extracted from licorice root under alkaline conditions showed greater anti-HIV activity than LCCs extracted by water [191]. Overall, these works show that extraction conditions play a significant role in the antiviral properties of LCCs.

There are also some reports addressing the antimicrobial and antiparasitic effects of LCCs. LCCs extracted from pine cones of *Pinus parviflora* via alkaline treatment have been reported to induce antimicrobial activity in mice infected with *Staphylococcus aureus*, a pathogen that causes a wide variety of clinical infections [196]. In another study, un-purified lignin extracted via alkaline extraction of corn stover residue, that was previously used for ethanol production, exhibited antimicrobial activities against the Gram-positive bacteria *S. aureus* and *Listeria monocytogenes* [197]. However, extracts did not show the same effect on Gram-negative bacteria such as *E. coli* and *S. enteritidis*. The study also indicated that the antimicrobial activities of the extracts were consistent with their antioxidant activities which were also affected by the extraction conditions (temperature and residue/solvent ratio).

LCCs also have been used for antitumor research. Based on folkloric information that the hot water extracts of pine cones is effective for gastroenterological tumors, Sakagami et al. investigated the antitumor activity of LCCs isolated from hot water and alkaline extracts of pine cones [198]. They found evidence that isolated LCCs significantly prolonged the survival of mice that had been implanted with ascites tumor cells (sarcoma-180). Another traditional medicine, *Inonotus obliquus* (Chaga mushroom), has been used to treat several human malicious tumors since the sixteenth century [199, 200]. Niu et al. investigated the characteristics and in vitro antioxidant and immunological activities of LCCs isolated from the alkaline extract of *I*. *obliquus* [200]. They indicated that extracts possessing multiple radical scavenging activities exhibited excellent antioxidant and immunological activities. These results suggest that some LCCs might be behind the legendary antitumor effects of some plants.

LCCs are also used as a natural UV blocking agent in sun creams and moisturizers. The anti-UV activity of resveratrol and vitamin C was compared with LCCs extracted from *Lentinus edodes* mycelia. The results showed that the anti-UV activity of LCCs was comparable to these two well-known UV-protective compounds [201]. In another study, LCCs extracted from pine cone and pine seed shell, by sequential alkaline extraction and acid precipitation processes, showed excellent anti-UV activity [202]. LCCs extracted from *Miscanthus sacchariflorus* (silvergrass) and *Pinus densiflora* (Japanese red pine) have been blended with a commercial cream, and their UV protection performance showed that SPF values increased in proportion to their amount [203]. Likewise, Ratanasumarn and Chitpraset studied the UV protection potential of lignin extracts (contains carbohydrate impurities) from alkaline-treated sugarcane bagasse and reported that the extract provided broad-spectrum UVA/UVB protection [204]. These results suggest a promising early commercial application of LCCs as personal care products, while pharmaceutical applications, and regulatory approval, can be established in the longer term.

### Biodegradable hydrogels and other drug delivery materials

Hydrogels are commonly defined as hydrophilic polymers providing a three-dimensional network which can contain a large volume of water. Advantageous properties generally include non-toxicity, high drug-loading capacity, biodegradability and biocompatibility, excellent support scaffold, and oriented architecture [205]. Hydrogels with these properties, have potential application in personal hygiene products, drug release devices, wound healing dressings, and regenerative medicines [206–208]. In recent years, the use of natural polymers has become increasingly popular for hydrogel development [209]. Hyaluronic acid, chondroitin sulfate, chitosan, gelatine, alginate, and cellulose derivatives have all been used to develop biopolymer-based hydrogel systems [210]. Lignin has a similar potential to be employed in the synthesis of biodegradable hydrogels; it contains many functional hydrophilic and functional groups (hydroxyls, carbonyls, methoxyls) allowing easy chemical modification for different applications [211]. Lignin has a number of inherent advantages such as antimicrobial, antioxidant, and biodegradable properties [209, 212]. Consequently, lignin-based hydrogels present promising properties as medical material coatings [23].

Cross-linking copolymerization, cross-linking of reactive polymer precursors, and cross-linking via polymer–polymer reaction are the three main methods used for synthesizing lignin-based hydrogels [213]. The main synthetic strategies and cross-linkers used to develop lignin-based hydrogels have been reviewed elsewhere [211]. Biocompatible hydrogels have been prepared by mixing chitosan solution (2.5%, w/v) in acetic acid with 10% (w/v) alkali lignin solution, and the resulting gels showed no cytotoxicity towards tested stem cells and animals; the authors concluded that the cross-linked products have great potential in wound healing applications [214]. By using a high amount of lignosulfonate and Al^+3^, Mondal et al. have prepared a hydrogel which exhibits ultrafast self-healing and outstanding antibacterial properties [215]. In another study, the mechanical stability and biocompatibility of hyaluronan-Kraft lignin-based hydrogels cross-linked with carbodiimide were tested. The authors reported that addition of Kraft lignin up to 3% (w/w) improved the resistance of the hydrogels [216]. Raschip et al. prepared hydrogel films by incorporating lignin extracted from annual fiber crops in xanthan gums (a common food additive and thickening agent) for vanillin release; they found that the lignin acted as an antioxidant agent and could improve compatibility and biocompatibility of the resultant hydrogels [217]. Recently, soluble lignin fractionated and isolated from the black liquor oil of empty fruit bunches by an acidification procedure was used to synthesize lignin-agarose hydrogel with epichlorohydrin as the cross-linking agent [208]. The study reported that the produced hydrogels have good mechanical properties. In another study, hydrogels have been synthesized through the radical polymerization of hardwood Kraft lignin and compared with synthetic hydrogels. This study showed that lignin-based hydrogels have a higher swelling capacity and are more thermally stable than synthetic hydrogels [218]. Lignin-based hydrogel development remains a developing research area, however, and there are limited therapeutic studies.

Apart from hydrogels, lignin-based nanoparticles (LNPs) can also be used to carry an active substrate. Due to their exceptional absorption capacity, biodegradability, and their non-toxic properties, LNPs have great potential for drug delivery [219]. Recent studies have shown that processes such as precipitation, solvent exchange, dialysis, and ultrasound can be applied to prepare drug-loaded nanoparticles [220]. LNP synthesis and characterization methods, as well as their advantages, have been recently reviewed [221] as has the impact of these processes on particle morphologies, drug loading capacities, and their potential applications [220]. LNPs have shown enhanced antioxidant and UV barrier properties compared to macromolecular lignin-based particles, and they have been tested for the release of silver ions used in cancer treatment [222–224]. They have also been evaluated for the delivery of doxorubicin hydrochloride (DOX), another anticancer drug which is used to treat many types of cancer such as leukemia, lymphoma, neuroblastoma, breast, and ovarian cancer. A recent study demonstrated the higher anticancer efficacy of DOX-loaded folic magnetic-functionalized LNPs [219].

A large number of pharmaceutical products are taken in solid dosage forms such as tablets. Excipients are the major components of these solid forms and are included to aid the manufacturing process or to add functionality to the drug compound [225]. A limited number of studies have tested the potential of lignin as an excipient and have shown that addition of lignin as an excipient can improve the drug release profile. In one recent study, lignin combined with microcrystalline cellulose was used as an excipient to prepare directly compressed tetracycline tablets; results showed that the presence of lignin in the tablets significantly modified the release profile and enhanced the amount of tetracycline released [224]. In another study, aspirin tablets containing lignin showed a higher release rate of the active pharmaceutical ingredient compared to the tablets without [226].

It is now clear that polymeric lignin, in various forms, has significant potential in the area of drug delivery and controlled release although further clinical evaluation is required.

## Conclusions and priorities for future study

The production of additional high value-added products from the lignin fraction of lignocellulosic biomass has the potential to enhance the economic viability of lignocellulosic biorefineries (Fig. 3). Lignin-based pharmaceutical production is an emerging strategy in biorefinery process design and has gained considerable attention recently. This review has shown that lignin-derived pharmaceuticals can be used to treat various important diseases such as cancer and diabetes. Some monolignols, specifically vanillin and ferulic acid, may be used as therapeutic agents against breast cancer, the most frequently diagnosed cancer among women. These monolignols are also capable of controlling blood glucose levels, vascular dysfunctions, and neurodegeneration associated with diabetes which is a major public health problem worldwide. Scientific evaluation of some “folk medicines” shows that the excellent antioxidant and immunological activities of LCCs might be the main cause of the legendary antitumor activities of some plant extracts. Furthermore, the broad antiviral effects of some lignin-derivatives have shown that in the close future, lignin can be used to treat life-threatening viral diseases such as HIV.

While broad therapeutic potential has been demonstrated, this review indicates that four major engineering and clinical hurdles remain before lignin-derived pharmaceuticals can be exploited commercially. These are summarized and discussed below.

- **Availability and processing.** The various sources of lignin (Section 2), and the impact of different pretreatment methods (Section 3), complicate the search for a common process technology that can reliably and predictively release defined products such as vanillin, ferulic acid, and eugenol. Further work is needed on milder and environmentally friendly pretreatment methods, such as the use of ionic liquids and deep eutectic solvents, to facilitate selective lignin extraction from lignocellulosic biomass with minimal degradation or by-product formation. Specifically, more studies focusing solely on pharmaceutical release from biorefinery waste streams are needed.
- **Isolation and characterization of defined products.** For pharmaceutical and therapeutic studies, products isolated from lignin or lignocellulosic biomass need to be well characterized in order to meet regulatory requirements. Here, further research on methods for the large-scale isolation of lignin-derived therapeutics is needed as well as on the characterization and quantification of product and impurity profiles as a basis for regulatory approval.
- **Yield and productivity improvement.** To produce high-value pharmaceuticals from lignin-based feedstock economically, the yield of the desired product (or intermediate) needs to be increased, and the synthesis of the final pharmaceutical (if required) has to be efficient. Further studies focusing on improving the yield and productivity of processes for waste lignin to pharmaceutical products are required alongside technoeconomic and life cycle evaluations to ensure financial and environmental sustainability.
- **Progression to human clinical trials.** Finally, in the majority of studies reported in this review, animal-based models have been used to evaluate the clinical potential of lignin and its derivatives, especially for vanillin, ferulic acid, and coumaric acid and for LCCs. Further clinical research with human subjects is needed to evaluate the true therapeutic potential of lignin-based products. Ideally, these need to be conducted with products released and purified from biomass sourced from an existing commercial biorefinery.

## Data Availability

Not applicable
